# Propolis-loaded nanostructured lipid carriers halt breast cancer progression through miRNA-223 related pathways: an in-vitro/in-vivo experiment

**DOI:** 10.1038/s41598-023-42709-7

**Published:** 2023-09-21

**Authors:** Sara A. Shaker, Shadi M. Alshufta, Mennatallah A. Gowayed, Noha S. El-Salamouni, Samar M. Bassam, Magda A. Megahed, Rasha A. El-Tahan

**Affiliations:** 1 Department of Biochemistry, Medical Research Institute, Alexandria University, Alexandria, Egypt; 2 Department of Clinical Pathology, Faculty of Medicine, Aden University, Aden, Yemen; 3 Department of Pharmacology and Therapeutics, Faculty of Pharmacy, Pharos University in Alexandria, Canal El-Mahmoudia Str., Smouha, Alexandria, Egypt; 4 Department of Pharmaceutics and Pharmaceutical Technology, Faculty of Pharmacy, Pharos University in Alexandria, Alexandria, Egypt; 5 Department of Pharmacognosy and Natural Products, Faculty of Pharmacy, Pharos University in Alexandria, Alexandria, Egypt

**Keywords:** Breast cancer, Cancer therapy, Pharmacology

## Abstract

The most frequent malignant tumor in women is breast cancer, and its incidence has been rising every year. Propolis has been used for its antibacterial, antifungal, and anti-inflammatory properties. The present study aimed to examine the effect of the Egyptian Propolis Extract (ProE) and its improved targeting using nanostructured lipid carriers (ProE-NLC) in Ehrlich Ascites Carcinoma (EAC) bearing mice, the common animal model for mammary tumors. EAC mice were treated either with 5-fluorouracil (5-FU), ProE, ProE-NLC, or a combination of ProE-NLC and 5-FU. Their effect on different inflammatory, angiogenic, proliferation and apoptotic markers, as well as miR-223, was examined. ProE and ProE-NLC have shown potential anti-breast cancer activity through multiple interrelated mechanisms including, the elevation of antioxidant levels, suppression of angiogenesis, inflammatory and mTOR pathways, and induction of the apoptotic pathway. All of which is a function of increased miRNA-223 expression. The efficiency of propolis was enhanced when loaded in nanostructured lipid carriers, increasing the effectiveness of the chemotherapeutic agent 5-FU. In conclusion, this study is the first to develop propolis-loaded NLC for breast cancer targeting and to recommend propolis as an antitumor agent against breast cancer or as an adjuvant treatment with chemotherapeutic agents to enhance their antitumor activity and decrease their side effects. Tumor targeting by ProE-NLC should be considered as a future therapeutic perspective in breast cancer.

## Introduction

Breast cancer has been the most popular malignant tumor in women, and its prevalence has been rapidly increasing^1,2^. The age of disease onset has been decreasing and there has been approximately a 14 percent increase in the mortality rate in the previous 5 years^3^. Chemotherapy, as well as radiotherapy, has several unwanted side effects, while cancerous cells are also usually resistant to therapy^4^. Reactive oxygen species (ROS) are known to induce oxidative damage to cell lipids and membranes, nucleic acids, and proteins; eventually leading to several chronic diseases such as atherosclerosis, diabetes, cancer, and other degenerative disorders^5–7^. Several studies recommended a combination of antioxidants as a possible potent preventive, adjunctive treatment for cancer^8,9^.

Lately, considerable attention has been given to the use of natural compounds for cancer treatment. Among these compounds, propolis (honeybee glue) is a resinous product that has been used as a health food and popular folk medicine for its antibacterial, anti-inflammatory, antioxidative, immunostimulatory, and carcinostatic properties^10^. However, propolis is known for its reduced solubility and poor bioavailability that hinder its efficacy. Nowadays nanotechnology has been extensively utilized to enhance the efficacy and reduce the toxicity associated with the use of common chemotherapeutics.

In this concern, this work aimed to utilize nanostructured lipid carriers (NLC) to overcome these challenges. Among several lipid-based nanocarriers, NLC proved to be superiorly effective for anti-cancer drug delivery compared to nano-emulsions that are subjected to phase separation problems and liposomes that may suffer from instability problems. Moreover, they offer superior drug loading capacity, enhanced stability, controlled drug release, and easier large-scale production^11–14^.

NLC are biodegradable, biocompatible nanocarriers that have been extensively reported to sustain the release and enhance the bioavailability as well as the therapeutic efficacy of several anti-breast cancer drugs^15–18^.

Based on the above-mentioned benefits of NLC for breast cancer targeting, the present study aimed to prepare and evaluate Propolis Extract (ProE) loaded NLC for improved targeting to different inflammatory, angiogenic, proliferation, and apoptotic markers as well as, mTOR and miR-223 related pathways in Ehrlich Ascites Carcinoma (EAC) bearing mice. To our knowledge, this study is the first to develop propolis-loaded NLC as a novel delivery system for breast cancer targeting, with excellent physicochemical properties and good stability, and to explore its efficacy when used alone or as adjuvant therapy.

## Materials and methods

### Materials

Precirol^®^ ATO 5 (P ATO 5), was gifted by Gattefosse, France. Castor oil, Morgan Specialty Chemicals, Cairo, Egypt. Miglyol^®^ 812 N, was a kind gift from Medizen Pharmaceutical Industries, Alexandria, Egypt. Kolliphor^®^ P 188 (Pluronic F68; P F68) and Kolliphor^®^ P 407 (Pluronic F127; P F127), were obtained as gift samples from BASF, Germany. Visking^®^ dialysis membrane size 36/32, 24 mm, MWCO 12,000–14,000; was obtained from Serva, Germany. Propolis was purchased from an Egyptian bee breeder in Damnhour, Egypt. Authentic reference materials for colorimetric assay were purchased from Sigma-Aldrich. All other chemicals were of analytical grades.

### Preparation and characterization of propolis extract (ProE)

#### Extraction of Egyptian propolis

An amount of 107 g of the finely pulverized propolis was macerated in 300 ml of 80% Ethanol. The mixture was shaken for 48 h on a mechanical shaker (130 rpm). After 48 h, the extract was filtered using filter paper and refrigerated. The remaining propolis residue was re-macerated in 300 ml 80% Ethanol for another 48 h on a shaker. The steps were repeated one more time. All the extracts were combined and evaporated to dryness using the rotavap^19^. A semisolid concentrated extract was obtained, not a solid residue. This was due to the wax content of the sample. Therefore, two methods were investigated to remove the wax and determine the method of choice.

#### Dewaxing

The traditional method of extract dewaxing was followed^20,21^ by filtering through Whatman paper No. 4. Later, the sample was kept in the fridge at 4 °C for 24 h and filtered again through Whatman paper No. 1. The resulting extract was dried using rotary and later freeze dried. However, it had poor solubility in ethanol and failed to provide a nanoformulation. Therefore, another method was investigated. Extract was kept at − 18 °C for 48 h, to allow agglomeration of wax, and then centrifuged for 2.5 min at 3500 rpm. The supernatant was separated and dried using rotavap followed by freeze-drying. This method yielded an extract with better solubility (10 folds) and a nanoformulation was successfully obtained. A 100 g crude propolis sample yielded around 4.5 g of dewaxed propolis extract. Wax comprised a great proportion of the crude sample. The UV spectrum showed a typical flavonoid spectrum with the characteristic two bands, Wavelength 283 nm was chosen for quantitative analysis, and a successful calibration curve was obtained using this method of propolis extract.

### Total phenolic and flavonoid contents determination

The standard used for total phenolic content determination was gallic acid, however, rutin was used for total flavonoid content determination. A stock solution was initially prepared by dissolving gallic acid in methanol at a concentration of 1 mg/ml, which was further diluted to offer concentrations of 50, 100, 200, 400, 600, 800 and 1000 µg/ml. Same stock solution was prepared for rutin, however, diluted to 10, 50, 100, 200, 400, 600 and 1000 µg/ml concentrations. For the total phenolic content, sample concentration was 2.5 mg/ml in methanol and for total flavonoid content it was 5 mg/ml in methanol. A microplate reader FluoStar Omega was used for recording results at 630 nm for gallic acid and 420 nm for rutin; after pipetting samples and standards in 6 replicates.

### LC- HR-MS/MS

#### Sample preparation

The ethanol extract was reconstituted with a solvent working solution prepared using (DI-Water: Methanol: Acetonitrile—50:25:25), the ratio of solvent to sample is 1 ml for 50 mg. The sample was shaken for 2 min followed by ultra-sonication for 10 min and centrifuged for 10 min at 10,000 rpm. An aliquot of 50 µl stock was diluted with 1000 µl reconstitution solvent. The final injected concentration was 1 µg/µl. Ten µl were injected in both modes. A volume of 10 µl of reconstitution solvent was injected as a blank sample.

#### Device and method

Analysis was performed on a high flow HPLC Exion LC column combined to an AB Sciex Triple TOF 5600 + mass spectrometer. The dimensions of the RP column were 2.1 × 50 mm, 3.5 µm, pre-handling was performed by means of “Phenomenex” pre-column and in-line filtration. Gradient elution at 0.3 ml/min and 40 °C was carried out using either 5 mM ammonium formate buffer (pH 8) containing 1% methanol or acidified deionized water (0.1% formic acid) with gradual increments of acetonitrile; according to the mode. Sample was developed for 28 min. Detection range was from 50 to 1000 Da. Nitrogen gas was used as a nebulizing, curtain and drying gas in MS1 acquisition. Collision energy of 35 V was used for MS2 acquisition with a spread of 20 V. The range for de-isotoping is 2 Da. Up to 15 ions were detected per cycle.

### Preparation of propolis-loaded NLC (ProE-NLC)

Propolis-loaded nanostructured lipid carrier (ProE-NLC) formulations were prepared by the modified high-shear homogenization method. Propolis extract (0.5%w/w) was dissolved in 100 µl ethanol by sonication, then dispersed homogenously in the molten lipid phase (4%w/w) in a water bath at 5 °C (Wise Bath, Seoul, Korea) above the lipid melting point.

The aqueous phase, containing the emulsifier was heated simultaneously to the same temperature as the lipid phase, poured onto it, and stirred for 1 min at 600 rpm (Snijders Magnetic stirrer, Holland). Then the mixture was homogenized for 5 min (Ultra Turrax T25, IKA, Staufen, Germany) at 15,000 rpm. The obtained NLC dispersion was allowed to cool at room temperature, then stored in the refrigerator at 4 °C for further characterization. Placebo formulations, free from ProE were prepared similarly^16^. The composition of the prepared NLC is shown in Tables 1 and 2.

**Table 1:** Entrapment efficiency of propolis extract-loaded nanostructured lipid carrier (ProE-NLC) with a different lipid composition.

Formulation	Lipid composition (%w/w)			EE (%)
	Solid lipid	Liquid lipid		
	P ATO 5	Castor oil	Miglyol	
F1	3.5	0.5	–	84.99 ± 0.87
F2	3	1	–	84.17 ± 2.61
F3	2.5	1.5	–	82.46 ± 0.64
F4	3.5	–	0.5	82.35 ± 0.35
F5	3	–	1	87.70 ± 2.82
F6	2.5	–	1.5	79.09 ± 1.89

All formulations contained 0.5% w/w propolis extract and 1%w/w Poloxamer 188.

**Table 2:** Characterization of optimized Pro-NLC formulations.

Formulation code	Emulsifier (%w/w)		Physicochemical properties		
	P 188	P 407	PS (nm)	PdI	EE (%)
F5	1	–	400.3 ± 2.25	0.398 ± 0.005	87.70 ± 2.82
F7	2	–	342.5 ± 1.62	0.362 ± 0.012	82.20 ± 0.85
F8	–	2	255.8 ± 0.67	0.263 ± 0.009	86.65 ± 1.60
F9	1	1	336.4 ± 1.47	0.205 ± 0.004	84.41 ± 0.43
F10	–	3	188.3 ± 0.23	0.267 ± 0.007	85.37 ± 0.69

All formulations contained 0.5% w/w propolis extract.

*P188* Poloxamer 188, *P407* Poloxamer 407, *PS* particle size, *PdI* polydispersity index, *EE* entrapment efficiency.

### In-vitro characterization

#### Particle size and polydispersity index

Particle size (PS) and polydispersity index (PdI) of ProE-NLC were determined using Zetasizer, (Malvern, UK). Diluted samples were measured in a glass cuvette with a square aperture at measurement position (4.65 mm) and 1.330 dispersant refractive index at 25 °C. For zeta potential (ZP) analysis, clear disposable zeta cells were utilized. The electrophoretic mobility of the sample was determined at 25 °C to calculate the ZP (DTS version 4.1 software, Malvern, UK). Samples were measured in triplicates and results were expressed as mean value ± SD.

#### Entrapment efficiency

The % entrapment efficiency (EE) of propolis in the NLC formulations was assessed by determining the un-entrapped propolis. Briefly, a definite volume (1 ml) of the prepared formulation was centrifuged at 15,000 rpm and 4 °C for 30 min (Hermle Z 32, Germany). The clear supernatant was suitably diluted by water, and the un-entrapped propolis was determined spectrophotometrically (λ_max_ 283 nm).

The % EE of propolis was calculated in triplicates using the equation below;

$${\text{EE }}\left( \% \right) \, = \, [({\text{W}}_{{{\text{total}}\,{\text{ProE}}}} {-}{\text{ W}}_{{{\text{free}}\,{\text{ProE}}}} )/{\text{W}}_{{{\text{total}}\,{\text{ProE}}}} ] \, \times { 1}00,$$

where W_total ProE_ is the total propolis amount in the prepared formulation and W_free ProE_ is the un-entrapped propolis amount (in the dispersion medium)^16^.

#### Surface morphology

The transmission electron microscope (TEM) was used to examine the surface morphology of the selected ProE-NLC formulation by the negative staining method. A drop of the diluted formulation on a carbon-coated copper grid was stained by 2% w/v uranyl acetate in ethanol, dried, and observed by TEM under suitable magnification (JEM-1400S plus, Joel, Tokyo, Japan)^22^.

#### In-vitro propolis release

The in-vitro propolis release from the ProE-NLC formulation was determined using the dialysis bag technique and compared to propolis dispersion. The dialysis bags, were pre-soaked overnight in distilled water, tied at one end, and filled with 0.5 ml of the tested formulation then, tied at the other end. The bags were placed separately in glass screw-capped containers, incorporating 50 ml phosphate buffer (PBS), pH 7.4, and shaken at 50 rpm and 37 ± 0.5 °C in a thermostatically controlled shaking water bath. At pre-determined time intervals, 0.5 ml samples were removed and replenished by an equal volume of a pre-warmed, fresh release medium to preserve the sink condition. The % propolis released from the ProE-NLC formulation at different time intervals was spectrophotometrically analyzed (λ_max_ 283 nm), using PBS (pH 7.4) as blank^22^. The experiment was done in triplicate.

The mechanism of propolis release from the selected formulation was analyzed by applying the release kinetics models. The in-vitro release profile was fitted to; Zero order, First order, Higuchi, Korsmeyer-Peppas and Hixon-Crowell models^23^.

#### Stability testing

The selected formulation was stored for 9 months, at 4 °C in well closed glass container. The changes of PS, PdI and % EE with storage time were investigated^24,25^.

### In-vivo experiments

#### Animals

70 healthy Balb/c female mice (10–12 weeks of age) with an average weight of 20–25 g were used in this study. Mice were purchased from the animal house of the Medical Research Institute, Alexandria University, and housed ten per cage. All mice were exposed to the same constant environmental conditions, a 12:12 h light/dark cycle with free access to food and water. Animals received a standard diet ad libitum, containing a total metabolizable percentage of the energy of 60.4 carbohydrates, 10.6 fat, and 29 proteins, 15.88 kJ gross energy/g. The study was approved by the “Institutional Animal Care and Use Committee (IACUC)-Alexandria University, Egypt” (Approval No.: AU01219123122). Experiments were completed in exact accordance with the regulations and guidelines of Egypt’s Guide for the Care and Use of Laboratory Animals. The study is also in compliance with ARRIVE guidelines and the “National Research Council’s Guide for the Care and Use of Laboratory Animals”. Efforts were made to diminution the distress of mice during the experiments.

#### Induction of Ehrlich ascites carcinoma (EAC)

Mammary tumors were induced in 60 mice by implanting EAC cells (0.2 ml of 2 × 10^5^ tumor cells in saline) subcutaneously in the mammary fat pad of mice^26^. The tumor was then allowed to progress to reach a considerable size of (> 150 mm^3^) before initiating drug treatment.

#### Experimental design

The EAC mice were divided into six groups (n = 10) as follows: (i) untreated EAC mice receiving dimethyl sulfoxide (DMSO, 0.25 mg/g)^27^. (ii) EAC mice treated with 5-Fluorouracil (5-FU, 20 mg/kg/day)^28^. (iii) EAC mice treated with Propolis (4 mg/kg/day)^29^. (iv) EAC mice treated with free NLC (4 mg/kg/day). (v) EAC mice treated with ProE-NLC (4 mg/kg/day)^29^. (vi) EAC mice treated with ProE- NLC (4 mg/kg/day) and with 5-FU (20 mg/kg/day). In addition to (vii) healthy normal female mice (NC) receiving DMSO. All drugs were administered for 9 days intraperitoneal starting from day one when the tumor reached a considerable size till day 9 (the end of the experiment).

#### Determination of the total mammary tumor volume

Tumor volumes were determined at the beginning and at the end of the treatment period. At each time, both the longer (a) and shorter (b) diameters of the tumor were determined using a Vernier caliper, and the tumor volume was calculated as follows:

$$\left[ {{\text{tumor}}\,{\text{volume }}\left( {\text{V}} \right) \, = \, 0.{4} \times {\text{ a}}\left( {\text{b}} \right)^{{2}} } \right]^{{{27}}} .$$

In animals bearing tumors at more than one site, tumor volume was the sum of the volumes of these tumors.

#### Samples collection

At the end of the specified treatment period (9 days), mice were sacrificed after deep anesthesia using isoflurane. Then blood samples were collected via cardiac puncture. The EAC tumor tissues were collected, washed with ice-cold saline, blotted dry and weighed. All tissue samples were stored at − 80 °C till biochemical analysis. Animals’ bodies were frozen until incineration.

#### Serum samples

The blood samples were collected, left for 20 min at 4 °C, and centrifuged at 3000×*g* for 10 min using Hettich Zentrifugen Tuttlingen centrifuge to obtain serum for determination of alanine aminotransferase (ALT), aspartate aminotransferase (AST) activities, urea, creatinine and malondialdehyde (MDA) levels. Serum levels of urea, creatinine, ALT and AST were assayed using commercially available kits (Bio-Med Diagnostic, OR, USA). The MDA was determined to correspond to the method of Draper and Hadley. The tissue samples were heated with thiobarbituric acid (TBA) at low pH and the resulting pink chromogen has shown a maximum absorbance at 532 nm^30^.

#### EAC tissues

EAC tissues were excised, homogenized using PBS and divided into two parts. The total homogenate was used for the determination of MDA content according to the method of Draper and Hadley. Another part was centrifuged to obtain supernatant which was used for the determination of catalase (CAT), superoxide dismutase (SOD), reduced glutathione (GSH), and glutathione reductase (GR) activities. All of the above-mentioned parameters were assayed using conventional commercial kits (Bio-Med Diagnostic, OR, USA). A modified method by Lowry et al. was performed for the determination of protein content in the samples^31^. Detailed methodology of each measurement is provided in the Supplementary File 1.

A 30 mg of EAC tissue was used for total RNA extraction for the determination of p53, nuclear factor kappa B (NF-κB), vascular endothelial growth factor (VEGF), matrix metalloproteinase-9 (MMP-9), toll-like receptor 4 (TLR4), high mobility group box 1 (HMBG1), the mammalian target of rapamycin (mTOR) and miRNA-223 gene expression using quantitative real-time polymerase chain reaction (qRT-PCR).

#### Relative gene expression analysis using quantitative real-time polymerase chain reaction (qRT-PCR)

Quantitative analysis of p53, NF-κB, VEGF, MMP-9, TLR4, HMBG, mTOR and mRNA-223 expression in EAC tissue was performed using qRT-PCR. Tissues (30 mg) were used for the total RNA extraction using the RNeasy kit (Qiagen, MD, USA) according to the manufacturer’s instructions. The integrity and concentration of the extracted RNA were controlled using nanodrop. Reverse transcription was performed using the Maxime™ RT PreMix kit (iNtRON Biotechnology Inc., Korea) according to the manufacturer's instructions. The tissue expression was quantified in the cDNA by Bio-Rad CFX qPCR (Qiagen, MD, USA) using QuantiTect SYBR Green PCR Master Mix (Qiagen, MD, USA). The quantitative PCR amplification conditions were regulated as follows: initial denaturation at 95 °C (10 min) followed by 45 cycles of PCR for amplification (Denaturation at 95 °C for 20 s, annealing at 55 °C for 20 s, and extension at 70 °C for 15 s). Data were collected using Bio-Rad CFX Maestro version 2.3 (Bio-Rad Inc., CA, USA). The expression of genes was quantified relative to the expression of the reference gene (18S rRNA or U6) in the same sample by calculating and normalizing the threshold cycles (Ct) values of target genes to that of the reference genes using ΔΔCt method (Table 3)^32^.

**Table 3:** Primers used for the qRT-PCR amplification.

Gene name	Accession number	Primer	
p53	NM_030989.3	Forward	5′-TGAGTTCCCTCAGCCATTGCCT-3′
		Reverse	5′-GAGGTTTCCTCTGGTCCTGGTA-3′
NF-κB	NM_009045.5	Forward	5′-TGAGTTCCCTCAGCCATTGCCT-3′
		Reverse	5′-GAGGTTTCCTCTGGTCCTGGTA-3′
VEGF	NM_031836.3	Forward	5′-TGAGTTCCCTCAGCCATTGCCT-3′
		Reverse	5′-GAGGTTTCCTCTGGTCCTGGTA-3′
MMP-9	NM_013599	Forward	5′-TGAGTTCCCTCAGCCATTGCCT-3′
		Reverse	5′-GAGGTTTCCTCTGGTCCTGGTA-3′
TLR4	NM_021297	Forward	5′-TGAGTTCCCTCAGCCATTGCCT-3′
		Reverse	5′-GAGGTTTCCTCTGGTCCTGGTA-3′
HMBG	NM_010439	Forward	5′-TGAGTTCCCTCAGCCATTGCCT-3′
		Reverse	5′-GAGGTTTCCTCTGGTCCTGGTA-3′
mTOR	NM_020009.2	Forward	5′-AGAAGGGTCTCCAAGGACGACT-3′
		Reverse	5′-CAGGACACAAAGGCAGCATTG-3′
18 s rRNA (Reference gene)	NR_046237.2	Forward	5′-AGG GCTGCT TTTAAC TCT GGT-3′
		Reverse	5′-CCC CAC TTGATT TTG GAG GGA-3′

### Statistical analysis

Data were expressed as means ± SD (n = 10) and analyzed using ANOVA to compare the different groups, followed by the LSD test as the post hoc test. Pearson was used for the correlation study. P < 0.05 was considered as the significance limit for all comparisons. The data were analyzed using SPSS software package version 20.0 (SPSS Chicago, IL, USA), and graphs were drawn using excel.

### Ethics declarations

The current study was approved by the “Institutional Animal Care and Use Committee (IACUC)-Alexandria University, Egypt” (Approval No.: AU01219123122). Experiments were performed in exact accordance with the regulations and guidelines of Egypt’s Guide for the Care and Use of Laboratory Animals. The study is also in compliance with ARRIVE guidelines and the “National Research Council’s Guide for the Care and Use of Laboratory Animals”.

## Results

### Phytochemical investigation of propolis extract

#### Total phenolic and flavonoid contents

The extract showed a good total phenolic and flavonoid content as follows:

$${\text{Total}}\,{\text{phenolics }}\left( {{\text{Gallic}}\,{\text{Acid}}\,{\text{Equivalent}}} \right) \, = { 91}.{51}\,{\text{mg}}/{\text{g}}\,{\text{Extract}}$$

$${\text{Total}}\,{\text{flavonoids }}\left( {{\text{Rutin}}\,{\text{Equivalent}}} \right) \, = { 9}0.{72}\,{\text{mg}}/{\text{g}}\,{\text{Extract}}$$

#### LC–MS/MS analysis

In this study, HR-LC–MS/MS was done for the identification of the major compounds contributing to the activity. High-resolution masses, in addition to ms^n^ fragments, were compared to data from the literature, databases such as DNP, HMDB, and our in-house database.

**Figure 1:**
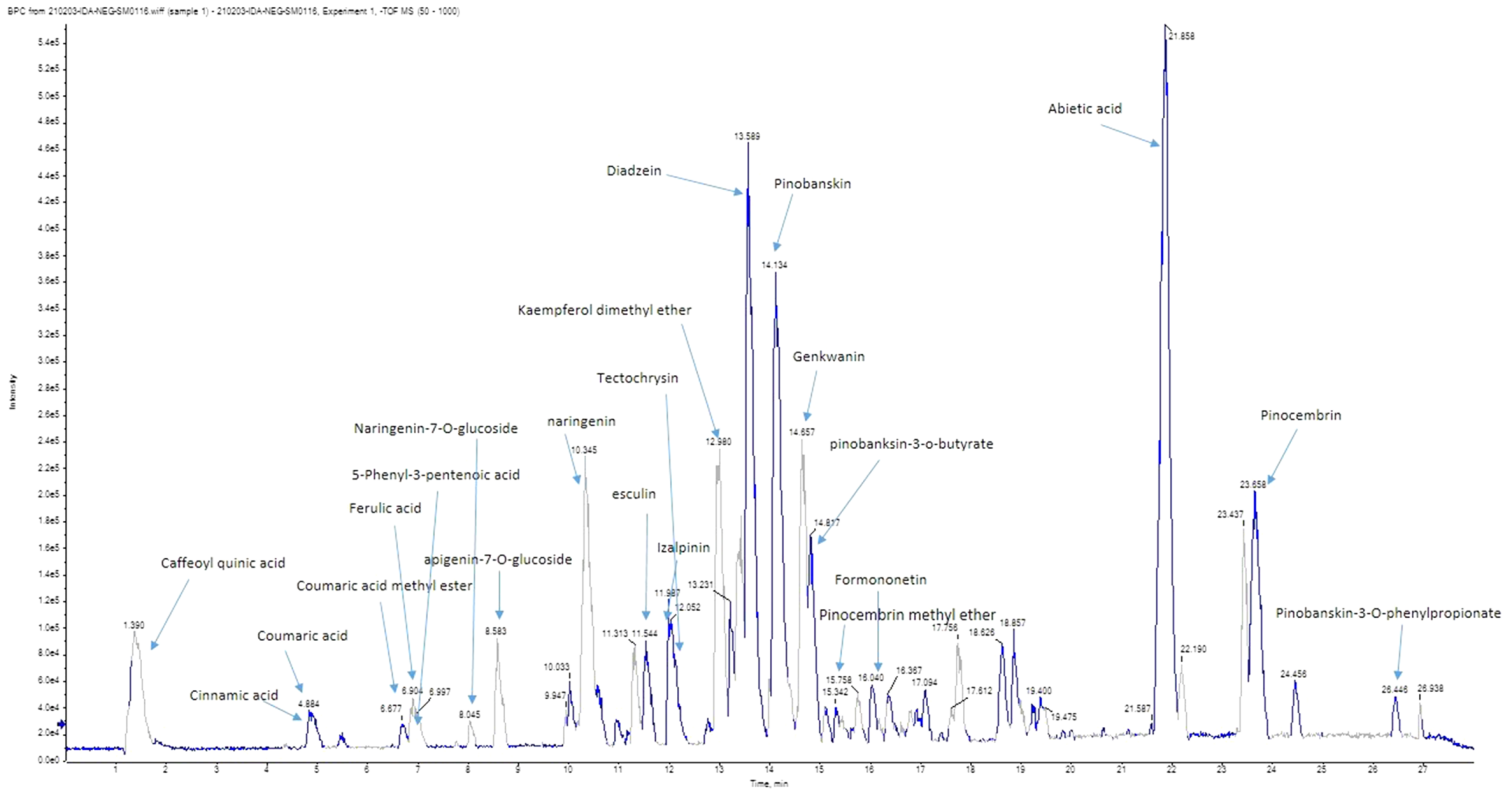
Base peak chromatogram of propolis in negative ion mode.

The analysis in the negative ion mode lead to the identification of 29 compounds represented in Table 4 and Fig. 1.

**Table 4:** LC–MS/MS analysis results.

	Rt	Measured *m/z* [M-H]^-^	Compound	Formula	MS/MS fragments	References
1	1.45	353.0835	Caffeoyl quinic acid	C16H18O9	191.0543, 172.936	^33^
2	4.81	147.0450	Cinnamic acid	C9H8O2	103.0542	^34^
3	4.86	163.0411	Coumaric	C9H8O3	nd	
4	6.77	177.0541	Coumaric acid methyl ester	C10H10O3	177.0542, 162.0315	
5	6.91	193.0505	Ferulic acid	C10H10O4	147.0405	^35^
6	7.32	175.0750	5-Phenyl-3-pentenoic acid	C11H11O2	nd	
7	8.01	433.1166	Naringenin-7-O-glucoside	C21H22O10	271.0452	^36^
8	8.10	435.1117	Phlorizin	C21H24O10	273.0789	^37^
9	8.11	193.0505	Isoferulic acid	C10H10O4	147.0405	^19^
10	8.50	431.0970	Apigenin-7-O-glucoside	C21H20O10	311.0601,269.0354	^33^
11	10.30	271.0616	Naringenin	C15H12O5	151.0036, 107.0140	^19^
12	11.39	339.0879	Esculin	C15H16O9	nd	
13	12.04	283.0622	Izalpinin	C16H12O5	268.0352, 240.0426	^19,38^
14	12.18	267.0655	Tectochrysin	C16H12O4	nd	^19,39^
15	13.00	313.0704	Kaempferol dimethyl ether	C17H14O6	298.283, 255.067	^40^
16	13.57	253.0504	Daidzein	C15H10O4	223.038,208.0501, 89.0006	^41^
17	14.10	271.0526	Pinobanskin	C15H12O5	253.09258	^40^
18	14.41	253.0502	Chrysin	C15H10O4	145.0292, 107.0137	^42^
19	14.55	283.0996	Phenethyl caffeate, CAPE	C17H16O4	nd	^40^
20	14.64	283.0623	Genkwanin	C16H12O5	269.04645, 151.0386	^19^
21	14.87	341.1008	Pinobanksin-3-o-butyrate	C19H18O6	nd	^40^
22	14.92	285.0449	Luteolin	C15H10O6	nd	^40^
23	15.12	269.0470	Pinocembrin methyl ether	C15H10O5	254.0618, 226.0617, 177.0180, 165.0200	^40^
24	15.80	269.0446	Galangin	C15H10O5	226.0617, 165.0200	^19^
25	16.04	267.0650	Formononetin	C16H12O4	nd	^43^
26	21.86	301.2182	Abietic acid	C20H30O2	nd	^19^
27	22.64	491.2438	Solophenol A or Nymphaeol C…prenylated polyphenol	C30H36O6	nd	^19^
28	23.66	255.2349	Pinocembrin	C15H12O4	213.0620, 151.0374	^19,40^
29	26.44	403.3063	Pinobanskin-3-*O*-phenylpropionate	C24H20O6	297.102, 271.054, 253.0500	^19,40^

*nd* ms^n^ fragments not detected.

### Preparation and preliminary optimization of propolis-loaded NLC

In preliminary study, different solid lipid types (Compritol^®^ 888 ATO and Precirol^®^ ATO 5) and concentrations (3, 4 and 6%) were studied to select the most suitable solid lipid to sufficiently encapsulate propolis and yield the smallest particle size. It was revealed that, using P ATO 5 yielded the smallest particle size, this is in accordance with previously reported results^44,45^. Thus, was selected as the solid lipid. Later, both Miglyol^®^ 812 N and castor oil, known to enhance encapsulation efficiency and impart better stability were studied as liquid lipids^46^.

As shown in Table 1, the prepared propolis-loaded NLC using different liquid lipid types and concentrations revealed ProE entrapment efficiency ranging from 79.09 ± 1.89 to 87.70 ± 2.82. The NLC formulation (F5), based on Miglyol (1%w/w) as liquid lipid revealed the highest EE of 87.70 ± 2.82%, thus, was chosen for further optimization.

The selected formulation has been prepared using Poloxamer 188, Poloxamer 407, or a mixture of both emulsifiers in different concentrations as shown in Table 2. The effect of emulsifier type and concentration on the PS, PdI as well as EE have been investigated. It was found that the NLC formulation stabilized by Poloxamer 407, showed a smaller size and PdI value (255.8 ± 0.67 and 0.263 ± 0.009, respectively) compared to that stabilized by Poloxamer 188 (342.5 ± 1.62 and 0.362 ± 0.012, respectively). Moreover, the increase in emulsifier concentration resulted in a significant decrease in particle size.

### Zeta potential

The ZP of the selected formulation revealed negatively charged particles (− 28.3 ± 4.71 mV).

### Surface morphology

**Figure 2:**
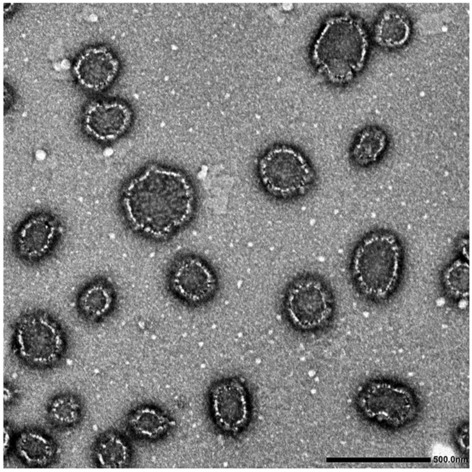
Transmission electron photomicrograph of the selected propolis-loaded nanostructured lipid carrier (ProE- NLC).

TEM revealed regular, spherical particles with a smooth surface with a diameter smaller than that revealed by photon correlation spectroscopy (175 nm revealed by TEM, compared to 188.3 nm by Malvern Zetasizer) as shown in Fig. 2.

### In-vitro propolis release

**Figure 3:**
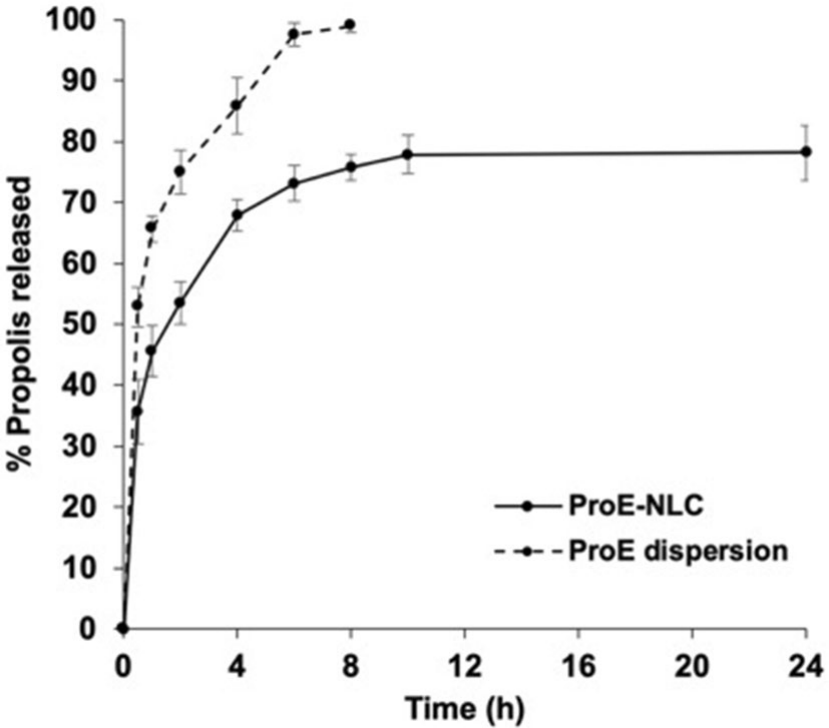
The in-vitro release profiles of propolis-loaded nanostructured lipid carrier (ProE- NLC) in phosphate buffer (PBS), pH 7.4, at 37 ± 0.5 °C. Results are presented as mean ± SD (n = 3).

As illustrated in Fig. 3, the optimized ProE-NLC formulation exhibited a significantly sustained release compared to ProE dispersion (*p* < 0.05). ProE dispersion profile showed a 100% release after 6 h, while, 80% of ProE was released within 24 h from the NLC formulation. ProE-NLC revealed a bi-phasic release pattern; showing a burst release of 45.56% during the first hour, followed by a sustained release of ProE over a period of 24 h. Model fitting showed that, ProE release from NLC followed Korsemeyer–Peppas model, (R^2^ = 0.9988). This model best described the sustained ProE release from NLC with n value; 0.301, indicating a Fickian mechanism of drug release (n ≤ 0.43). Thus, diffusion occurs in response to a concentration gradient.

### Stability testing

The physical stability of the selected NLC formulation was assessed after 9 months of storage at 4 °C (Table 5). Visually, the formulation remained unchanged without any signs of creaming, settling, or coalescence. A significant increase in the particle size was observed as a function of time (213.9 nm), compared to the freshly prepared formulation (188.3 nm). The PdI of the tested formulation has also increased with storage from 0.267 to 0.416.

**Table 5:** Effect of storage time at 4 °C on particle size, polydispersity index and entrapment efficiency of the selected ProE NLC.

Storage time (months)	PS (nm)	PDI	EE (%)
0	188.3 ± 0.23	0.267 ± 0.007	85.37 ± 0.69
3	190.9 ± 2.61	0.372 ± 0.010	85.40 ± 2.12
6	203.6 ± 3.82	0.402 ± 0.020	84.91 ± 1.77
9	213.9 ± 2.86	0.416 ± 0.004	84.28 ± 0.46

Values are represented as means ± SD, (n = 3).

*PS* particle size, *PdI* polydispersity index, *EE* entrapment efficiency.

On the other hand, no significant change in the amount of entrapped ProE was observed after storage (84.28% EE).

### In-vivo experiments

#### Tumor volume

**Figure 4:**
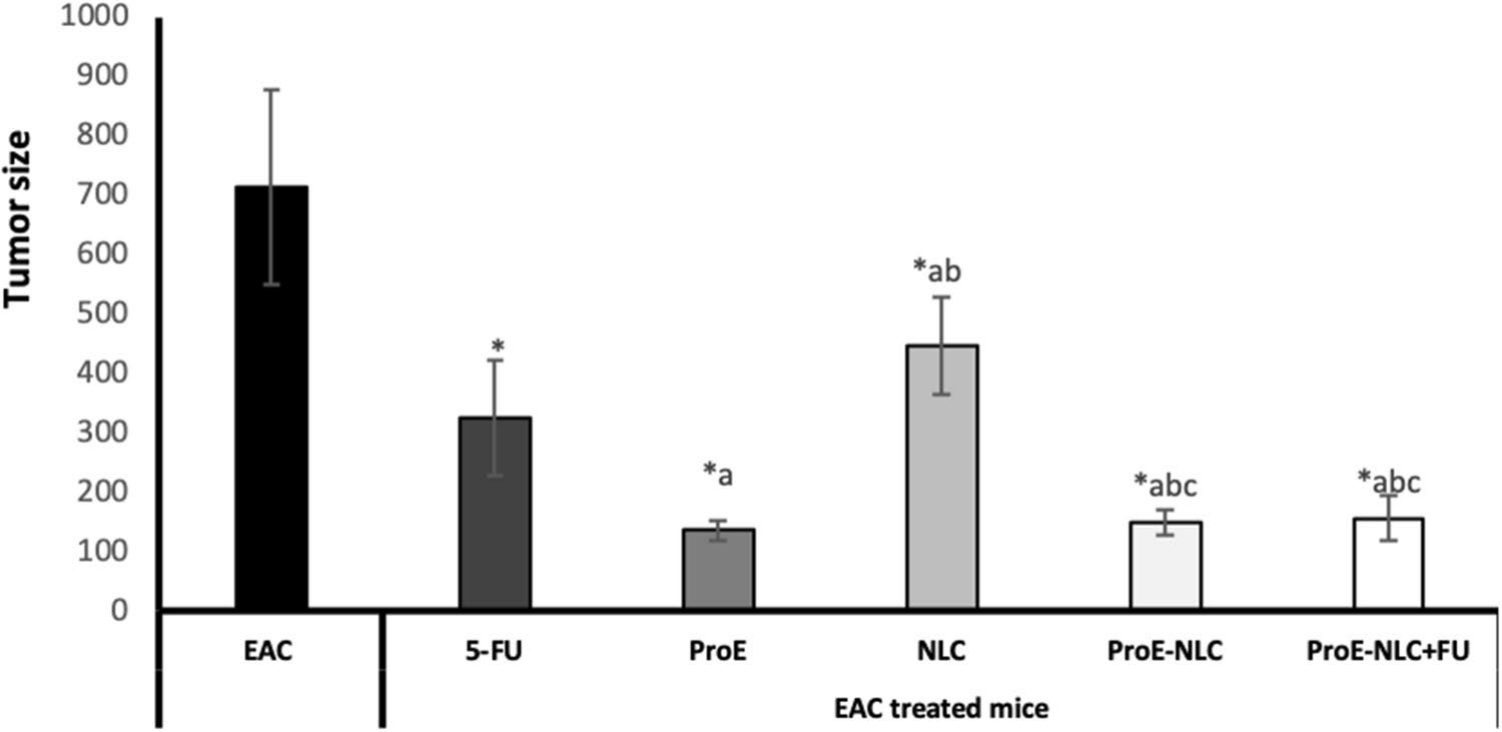
Tumor volume in all studied groups. Data are presented as means ± SD (n = 10). Comparisons among groups were analyzed using one-way ANOVA followed by an LSD post-hoc test. Data are compared at p < 0.05 with the EAC (*), F-FU (**a**), ProE (**b**), NLC (**c**) and ProE-NLC (**d**). *EAC* untreated Ehrlich ascites carcinoma, *5-FU* 5-Fluorouracil, *ProE* propolis extract, *NLC* nanostructured lipid carrier, *ProE-NLC* propolis extract-nanostructured lipid carrier.

The results of tumor volume are shown in Fig. 4. All EAC-bearing treated mice showed significantly lower tumor volume compared with untreated EAC-bearing mice (p ≤ 0.05). ProE alone or ProE-NLC and the combined group showed lower tumor volume compared to 5-FU and NLC treated groups (p ≤ 0.05).

#### Kidney and liver function tests

The results of serum urea and creatinine levels are shown in Table 6. The untreated EAC-bearing mice showed significantly higher urea and creatinine levels compared to the control group (p ≤ 0.05). All EAC-bearing treated mice showed significantly lower urea and creatinine levels compared with untreated EAC-bearing mice (p ≤ 0.05).

**Table 6:** Serum urea, creatinine levels and ALT, AST activities in all studied groups.

Groups		Urea (mg/dl)	Creatinine (mg/dl)	AST (U/l)	ALT (U/l)
NC		26.30 ± 4.05	0.219 ± 0.032	28.7 ± 5.49	32.7 ± 6.03
EAC bearing mice	EAC	42.10 ± 5.25^#^	0.328 ± 0.089^#^	286.2 ± 41.33^#^	135.0 ± 12.2^#^
	5-FU treated	37.80 ± 3.32^#,^*	0.267 ± 0.055*	163.4 ± 18.68^#,^*	134.200 ± 14.18^#^
	ProE treated	26.80 ± 3.32*^,a^	0.240 ± 0.025*	46.6 ± 10.44^#,^*^,a^	120.800 ± 9.1^#,^*^,a^
	NLC treated	40.0 ± 3.71^#,b^	0.339 ± 0.063^#,a,b^	63.1 ± 9.8*^,a,b^	87.2 ± 8.5^#,^*^,a,b^
	ProE-NLC treated	31.9 ± 4.06^#,^*^,a,b,c^	0.257 ± 0.033*^,c^	39.8 ± 6.6^#,^*^,a,b^	47.800 ± 7.51^#,^*^,a,b,c^
	ProE-NLC + 5-FU	27.1 ± 3.5^#,^*^,a,b,c,d^	0.259 ± 0.033*^,c^	35.3 ± 5.8^#,^*^,a,b^	57.4 ± 11.03^#,^*^,a,b,c,d^

Comparisons among groups were analyzed using one-way ANOVA followed by LSD test. Values are presented as means ± SD (n = 10). Data are compared at p < 0.05 with NC (#), EAC (*), 5-FU (a). ProE (b), NLC (c) and ProE-NLC (d). *NC* normal control, *EAC* untreated Ehrlich ascites carcinoma, *5-FU* 5-Fluorouracil, *ProE* propolis extract, *NLC* nanostructured lipid carrier, *ProE-NLC* propolis extract- nanostructured lipid carrier.

The results of alanine aminotransferase (ALT) and aspartate aminotransferase (AST) activities are shown in Table 5. The untreated EAC-bearing mice showed significantly higher ALT and AST activities compared to the control group. All EAC-bearing treated mice showed significantly lower ALT and AST activities compared with untreated EAC-bearing mice, except for mice treated with 5-FU showed no significant change in ALT activity compared to untreated mice. ProE-NLC treated mice showed lower ALT activity, however, the combined treated group showed lower AST activity compared to all treated groups (p ≤ 0.05).

#### Oxidative stress markers

The results of the oxidative stress marker, malondialdehyde (MDA), as well as the antioxidant markers, superoxide dismutase (SOD), catalase and glutathione reductase (GR), are shown in Table 7. The untreated EAC-bearing mice showed significantly higher MDA levels in serum compared to the control group (p ≤ 0.05). All EAC-bearing treated mice showed significantly lower serum levels and tissue contents of MDA compared with untreated EAC-bearing mice, except 5-FU treated mice showed significantly higher MDA in both serum and tissue compared to all treated groups (p ≤ 0.05). ProE-NLC treated mice showed lower serum and tissue MDA compared to all treated groups (p ≤ 0.05).

**Table 7:** Serum level and tissue content of MDA, solid tumor tissue SOD, catalase, GR and GSH activities, and TAC in all studied groups.

Groups		MDA (nmol/ml)	MDA (nmol/g tissue)	SOD activity (U/mg protein)	Catalase activity (U/mg protein)	GR activity (mU/mg protein)	rGSH activity (µmol/mg protein)	TAC (nmol/mg protein)
EAC bearing mice	EAC	55.250 ± 3.45^#^	105.200 ± 3.489	11.900 ± 1.116	12.300 ± 2.246	57 ± 2.7	39.210 ± 2.16	151 ± 3.3
	5-FU treated	64.29 ± 7.4^#,^*	130.000 ± 1.69*	11.000 ± 0.226	10.800 ± 0.503*	41.5 ± 1.2*	29.700 ± 1.22*	128.000 ± 3.4*
	ProE treated	23.29 ± 2.18^#,^*^,a^	87.600 ± 3.399*^,a^	31.500 ± 1.269*^,a^	26.110 ± 0.922*^a^	88 ± 2.26*^,a^	82.800 ± 1.77*^,a^	329.000 ± 1.9*^,a^
	NLC treated	36.45 ± 4.5^#,^*^,a,b^	97.500 ± 3.979*^,a,b^	16.800 ± 1.273*^,a,b^	17.400 ± 0.592*^,a,b^	51.5 ± 2.71*^,a,b^	45.210 ± 0.68*^,a,b^	158.000 ± 2.9*^,a,b^
	ProE-NLC treated	22.75 ± 4.03^#,^*^,b,c^	69.000 ± 2.260*^,a,b,c^	36.590 ± 1.537*^,a,b,c^	30.600 ± 0.287*^,a,b,c^	108.1 ± 3.34*^,a,b,c^	88.900 ± 1.76*^,a,b,c^	357.1 ± 2.7*^,a,b,c^
	ProE-NLC + 5-FU	26.43 ± 2.7^#,^*^,b,c^	89.500 ± 2.718*^,a,c,d^	26.900 ± 1.115*^,a,b,c,d^	24.010 ± 0.91*^,a,b,c,d^	87.5 ± 4.249*^,a,c,d^	74.8 ± 1.5*^,a,b,c,d^	297 ± 5.16*^,a,b,c,d^

Comparisons among groups were analyzed using one-way ANOVA followed by LSD post-hoc test. Values are presented as means ± SD (n = 10). Data are compared at p < 0.05 with EAC (*), F-FU (a). ProE (b), NLC (c) and ProE-NLC (d). *EAC* untreated Ehrlich ascites carcinoma, *5-FU* 5-Fluorouracil, *ProE* propolis extract, *NLC* nanostructured lipid carrier, *ProE-NLC* propolis extract-nanostructured lipid carrier, *MDA* malondialdehyde, *SOD* superoxide dismutase, *GR* glutathione reductase, *rGSH* reduced glutathione, *TAC* total antioxidant capacity.

All EAC-bearing treated mice showed significantly higher SOD and catalase activities compared with untreated EAC-bearing mice (p ≤ 0.05), except for 5-FU treated mice showed no significant difference compared with untreated EAC-bearing mice (p ≤ 0.05). Mice treated with the ProE-NLC group showed higher SOD and catalase contents compared to all treated groups (p ≤ 0.05).

Results of GR and reduced glutathione (rGSH) activities came to show that all EAC-bearing treated mice showed significantly higher GR and rGSH activities compared with untreated EAC-bearing mice (p ≤ 0.05), except for mice treated with 5-FU showed a significant decrease compared with untreated EAC-bearing mice (p ≤ 0.05). Mice treated with ProE-NLC showed higher GR and rGSH activities compared to all treated groups (p ≤ 0.05).

All EAC-bearing treated mice showed significantly higher total antioxidant capacity (TAC) compared with untreated EAC-bearing mice (p ≤ 0.05), except for mice treated with 5-FU showed significantly lower TAC compared with untreated EAC-bearing mice and all treated mice (p ≤ 0.05). ProE-NLC treated mice showed higher TAC levels compared to all treated groups (p ≤ 0.05).

#### Gene expression analysis

##### Inflammatory markers

**Figure 5:**
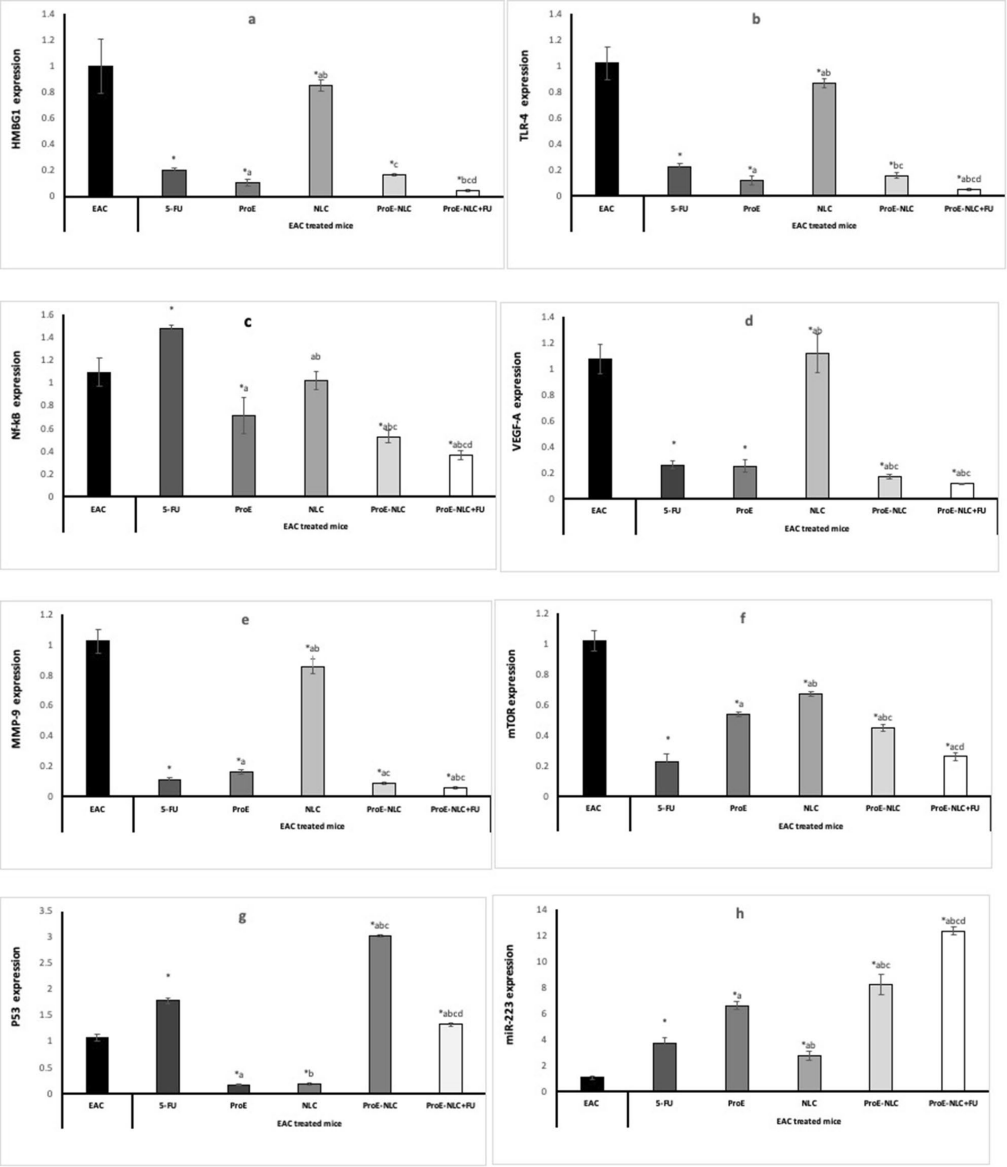
Gene expression of (**a**) high mobility group box 1 (HMBG1), (**b**) toll-like receptor 4 (TLR4), (**c**) nuclear factor kappa B (NF-κB), (**d**) vascular endothelial growth factor-A (VEGF-A), (**e**) matrix metalloproteinase-9 (MMP-9), (**f**) the mammalian target of rapamycin (mTOR), (**g**) p53, (**h**) miRNA-223. Data are presented as means ± SD (n = 10). Comparisons among groups were analyzed using one-way ANOVA followed by an LSD post-hoc test. Data are compared at p < 0.05 with EAC (*), F-FU (**a**), ProE (**b**), NLC (**c**) and ProE-NLC (**d**). *EAC* untreated Ehrlich ascites carcinoma, *5-FU* 5-Fluorouracil, *ProE* propolis extract, *NLC* nanostructured lipid carrier, *ProE-NLC* propolis extract-nanostructured lipid carrier.

The results of HMBG1, TLR4, and NF-κB expression are shown in Fig. 5a–c. All EAC-bearing treated mice have shown significantly lower HMBG, TLR4 and NF-κB expressions compared with untreated EAC-bearing mice (p ≤ 0.05), except for EAC-bearing mice treated with 5-FU, which showed significantly higher NF-κB expression compared with untreated EAC-bearing mice and all other treated mice. ProE-NLC with 5-FU treated mice showed the lowest HMBG, TLR4 and NF-κB expressions compared with all treated groups (p ≤ 0.05).

##### Angiogenic markers

The results of VEGF-A and MMP-9 expressions are shown in Fig. 5d,e. All EAC-bearing treated mice showed significantly lower expressions of VEGF-A and MMP-9 compared with untreated EAC-bearing mice (p ≤ 0.05). ProE-NLC with 5-FU treated mice showed the lowest expressions of VEGF-A and MMP-9 compared to all treated groups (p ≤ 0.05).

##### Proliferation marker

The results of mTOR expression are shown in Fig. 5f. All EAC-bearing treated mice showed significantly lower mTOR expression compared with untreated EAC-bearing mice (p ≤ 0.05). The EAC-bearing mice treated with 5-FU and ProE-NLC with 5-FU showed the lowest mTOR expression compared to all treated groups (p ≤ 0.05).

##### Apoptotic marker

The results of p53 expression are shown in Fig. 5g. All EAC-bearing treated mice showed significantly higher p53 expression compared with untreated EAC-bearing mice (p ≤ 0.05), except for EAC-bearing mice treated with 5-FU and NLC treated groups showed significantly lower p53 expression compared with untreated EAC bearing mice and all other treated mice (p ≤ 0.05). The ProE-NLC treated mice showed the highest p53 expression compared with all other treated groups (p ≤ 0.05).

##### miRNA-223

The results of miRNA-223 expression are shown in Fig. 5h. All EAC-bearing treated mice showed significantly higher miRNA 223 expression compared with untreated EAC-bearing mice (p ≤ 0.05). Combined treated EAC-bearing mice showed the highest miRNA-223 expression compared with all treated groups (p ≤ 0.05).

##### Correlation studies

**Figure 6:**
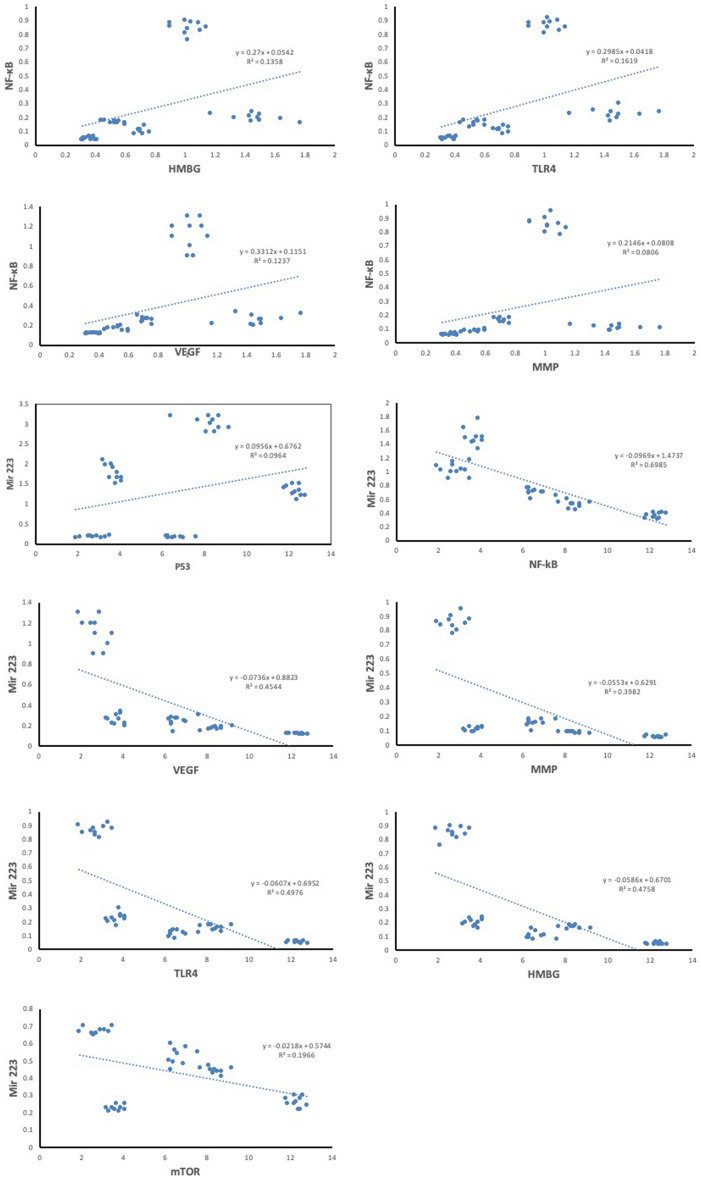
Correlation study between different gene expressions in EAC-bearing treated groups. *HMBG1* high mobility group box 1, *TLR4* toll-like receptor 4, *NF-κB* nuclear factor kappa B, *VEGF-A* vascular endothelial growth factor-A, *MMP-9* matrix metalloproteinase-9, *mTOR* the mammalian target of rapamycin.

The results of the statistical study among EAC-bearing treated groups using Pearson correlation (Fig. 6) showed that NF-κB expression is positively correlated with expressions of HMBG1 (r = 0.369, p = 0.003), TLR4 (r = 0.402, p = 0.004), VEGF-A (r = 0.352, p = 0.013) and MMP-9 expressions (r = 0.285, p = 0.048). The miR-223 expression was positively correlated with p53 expression (r = 0.310, p = 0.030) and negatively correlated with expressions of NF-κB (r = − 0.836, p = 0.001), VEGF-A (r = − 0.674, p = 0.001), MMP-9 (r = − 0.631, p = 0.001), TLR4 (r = − 0.705, p = 0.001), HMBG1 (r = − 0.690, p = 0.001) and mTOR expressions (r = − 0.443, p = 0.001).

## Discussion

It is known that miRNA-223 is being highly expressed in the tumor microenvironment^47^, however, its increased expression and its significance in breast cancer are poorly explored. This study came to show the role of increased expression of miRNA-223 in breast cancer suppression and its related pathways. Knowing that propolis extracts and their active chemicals are able to alter the cancer development processes, influence the tumor microenvironment, and are chemosensitizers of multidrug-resistant cancer cells, reducing the negative effect of chemotherapy and radiotherapy^48,49^, encouraged the use of the Egyptian propolis extract in the current study to enhance the expression of miRNA-223.

Results of LC–MS analysis of Egyptian ProE revealed the presence of twenty-nine chromatographic peaks belonging to different classes of compounds that were identified including flavonoids, phenolic acids, and fatty acids. Most of the ProE ingredients have various biological effects namely; antimicrobial effects against several bacteria, fungi, and viruses, antiparasitic, anti-inflammatory, antiproliferative, antioxidant, immunomodulatory, anti-carcinogenic properties and hepatoprotective effects^50,51^. These data correspond to the results of other studies investigating the constituents of ProE which showed that the phenolic content of propolis is mainly composed of daidzein, chrysin, abietic acid, galangin, pinobanksin and pinocembrin, the last being the most abundant flavonoid in propolis^52,53^.

NLC is considered a promising delivery system that offers improved bioavailability of the incorporated drugs and hence could be used to overcome the major drawback of low solubility and poor bioavailability of ProE^54^. After an extensive literature review, it was found that the anti-breast cancer efficacy of propolis extract has never been evaluated from propolis-loaded NLC^55–57^.

In a preliminary study, propolis-loaded NLC was prepared using different lipid compositions and evaluated for EE. Satisfactory results were revealed, attributed to the hydrophobic property of ProE. F5 showed the highest EE and thus was chosen to further evaluate the effect of emulsifier type and concentration on the NLC. It was found that, increasing the emulsifier concentration resulted in a significant PS decrease. Thus, F10 which showed the smallest PS (188.3 nm), low PdI (0.267) and satisfactory EE (85.37%) was selected for further in-vitro and in-vivo evaluation. Moreover, ProE-NLC was negatively charged suggesting a physically stable dispersion. Morphological examination revealed un-aggregated nanocarriers with a smaller size compared to zetasizer, due to the sample preparation procedure prior to the TEM examination.

Regarding the ProE-NLC release profile, the initial burst release might be due to the free un-entrapped drug (15%). However, the immobilization of the remaining ProE (85%) in the lipid matrix, resulted in a sustained release profile, where only 75.70% of the drug was released after 8 h. It might also be due to the faster release of ProE dissolved in the liquid lipid component of the NLC followed by a more sustained release of the drug in the solid lipid^25^. This busrt release could be useful to provide an initial theratpeutic effect of ProE followed by a controlled release of the drug entrapped in the nanoparticle core. Consequently, ProE-NLC is a promising carrier to sustain ProE release for breast cancer treatment. Moreover, the physical stability of ProE-NLC was confirmed after 9 months of storage, with a small particle size growth that might be attributed to the fusion of small particles^25,58^. However, it remained in a small nanometric size below 250 nm. Moreover, ProE entrapment was almost unchanged, most probably due to the the complex lipid core, composed of P ATO 5 as solid lipid and Miglyol as liquid lipid. Several studies proved that, complex lipid core results in imperfection in the crystal structure, allowing more space for drug accommodation and reduces the probability of drug expulsion during storage^59^. It is worth mentioning that the insignificant reduction in the entrapment efficiency upon storage from 85.37 ± 0.69 to 84.28 ± 0.46% after 9 months storage, might be due to the leakage of some the loaded drug as a result of the possible fusion of small particles to form larger ones.

Mice injected with EAC cells and treated with 5-FU, ProE, ProE-NLC, and combined ProE-NLC + 5-FU showed a constant body weight (data shown in a Supplementary File 2) with a significant reduction in the tumor volume compared to mice in the untreated EAC group. Such an outcome was associated with decreased oxidative stress and increased antioxidant markers in all treated groups. Key enzymes in the antioxidant defense mechanism are SOD and CAT, which are subjected to alteration during carcinogenesis^60^. This disequilibrium of the antioxidant defense system makes cells more exposed to free radicals. Decreased activities of the antioxidant enzymes cause an accumulation of ROS causing the destruction of DNA, RNA, proteins, and lipids ultimately affecting cancer cells. Lipid peroxidation is a catalytic propagating free radical chain reaction known for its association with pathological cell conditions, resulting in tissue degeneration. The last product of lipid peroxidation is MDA, which is found to be higher in tumor tissues over that in healthy tissues^53^, and was further confirmed in this study.

Both GSH and SOD are involved in the cell protection process against ROS and the development of cancer^61^. Sun et al.^62^ has shown an inhibition of SOD activity in EAC-bearing animals, an effect that may be attributed to the mitochondrial loss in EAC cells. This in turn results in lowering the total hepatic SOD activity. Moreover, the considerable reduction in CAT activity in the plasma of tumor-bearing mice came in agreement with analog findings observed by Bozzi et al.^63^. It is suggested that elevated levels of SOD, catalase, GR, GSH and TAC contents in EAC cells, and decreased MDA content obtained from ProE and ProE-NLC treated mice is attributed to the free radical scavenging activity of the flavonoids in the ProE^64–66^. The ability of ProE to decrease lipid peroxidation was also able to improve the kidney and liver biomarkers, agreeing with previous work that reduced hepatic enzyme levels in serum is one of the indications of the antitumor potential^67^.

Exploring the molecular pathway, results have shown that all EAC-bearing mice treated with ProE, ProE-NLC with/without 5-FU showed significantly higher miR-223 expression compared with untreated EAC-bearing mice. The miR-223 is an anti-inflammatory miRNA that in cancer acts either as an oncosuppressor or oncopromoter, in a context-dependent manner^68^, whereas in breast cancer, they dampened the activation of the EGF-associated cancerous pathway^69^. Such increased expression, especially by the combined ProE-NLC + 5-FU, was associated with decreased breast cancer cell proliferation and apoptosis, demonstrated as decreased mTOR expression and increased p53 expression. The p53 tumor-suppressor protein is a transcription factor that controls the rate of transcription of number of genes implicated in cell cycle regulation, DNA repair, and apoptosis^70^. While mTOR signaling regulates cell metabolism and proliferation responsible for tumor initiation and progression^71^. It has been proven that the active component of propolis, caffeic acid phenethyl ester, has pro-apoptotic activity through the caspase-3/7 pathway^72–74^. Chrysin, another component of propolis, has also a well-understood pro-apoptotic activity and molecular mechanism, initiating apoptosis via the mitochondrial pathway^67^. Pinobanskin^75^, daidzein^76^, abietic acid^77^ and other components of propolis have shown also the ability to protect against oxidative damage through Akt/mTOR pathway^78^.

Numerous studies demonstrated that inflammation plays a critical role in the tumorigenesis^79,80^ and^81,82^ and accumulating evidence indicated good antitumor and anti-inflammatory abilities of the propolis^83^. Our results indicated that all EAC-bearing treated mice showed significantly lower TLR4, HMBG1, and NF-κB expressions compared with untreated EAC-bearing mice, with the most prominent decrease in mice treated with a combination of ProE-NLC and 5-FU. The HMGB1 is a non-histone protein mostly localized in the cell nucleus. It interacts with DNA to stimulate the nuclear transcription processes. However, dead, dying, or injured cells can release HMGB1 passively into the extracellular matrix. It can also be secreted by cancer and immune cells in response to various exogenous or endogenous stimuli. Secreted HMGB1 acts as an “alarmin” or a “danger signal” that may trigger malignant tumor progression^84^. It was reported that HMGB1 can act as a ligand for several immune receptors like TLR-2 and -4^85^.

Toll-like receptors have gathered an extraordinary amount of interest in cancer research due to their role in the tumor progression^86^, where its activation has been linked to both cancer inhibition and growth. Yang et al.^87^ reported that ten TLRs were expressed in MDA-MB-231 cells, and TLR-4 expression was the highest among all the TLRs. Moreover, they demonstrated that the knockdown of TLR-4 could actively inhibit the proliferation and survival of breast cancer cells. The TLR4 signaling pathway includes MyD88-dependent and MyD88-independent pathways. Both pathways can activate NF-κB to release cytokine^88^. This explains the positive correlation between NF-κB with HMBG1 and TLR4 expressions in the current study. Chang et al. have shown that Chinese propolis and its major constituent—CAPE was able to control breast cancer cell proliferation and survival through the TLR-4/NF-κB signaling^83^, which supports the use of the Egyptian propolis in this study.

Experimental data show that propolis and some of its components have anti-angiogenic activity against neoplastic cells, decreasing the activation of hypoxia-inducible factor 1 (HIF-1α), and hence reducing vascularization induced by VEGF and consequently suppressing tumor growth. Propolis was able to directly inhibit VEGF production, as well as inhibit MMP-2 and -9 production^89^. The upregulation of VEGF and MMPs, especially MMP-9, in breast cancer are well known and are considered responsible for tumor invasion and metastasis^90,91^. It has been suggested that the anti-angiogenic activity of propolis concerns the downregulation of the activity of cell signaling pathways mediated by Jun N-terminal kinase, ERK1/2, NF-κB, Akt, and PAK1 pathways^74,92,93^, where NF-κB regulates the expression of various molecules important in tumorigeneses, such as MMPs, VEGF-A and inflammatory cytokines^94^. This aligns with the positive correlation between NF-κB with MMP9 and VEGFA expressions in this study.

## Conclusion

**Figure 7:**
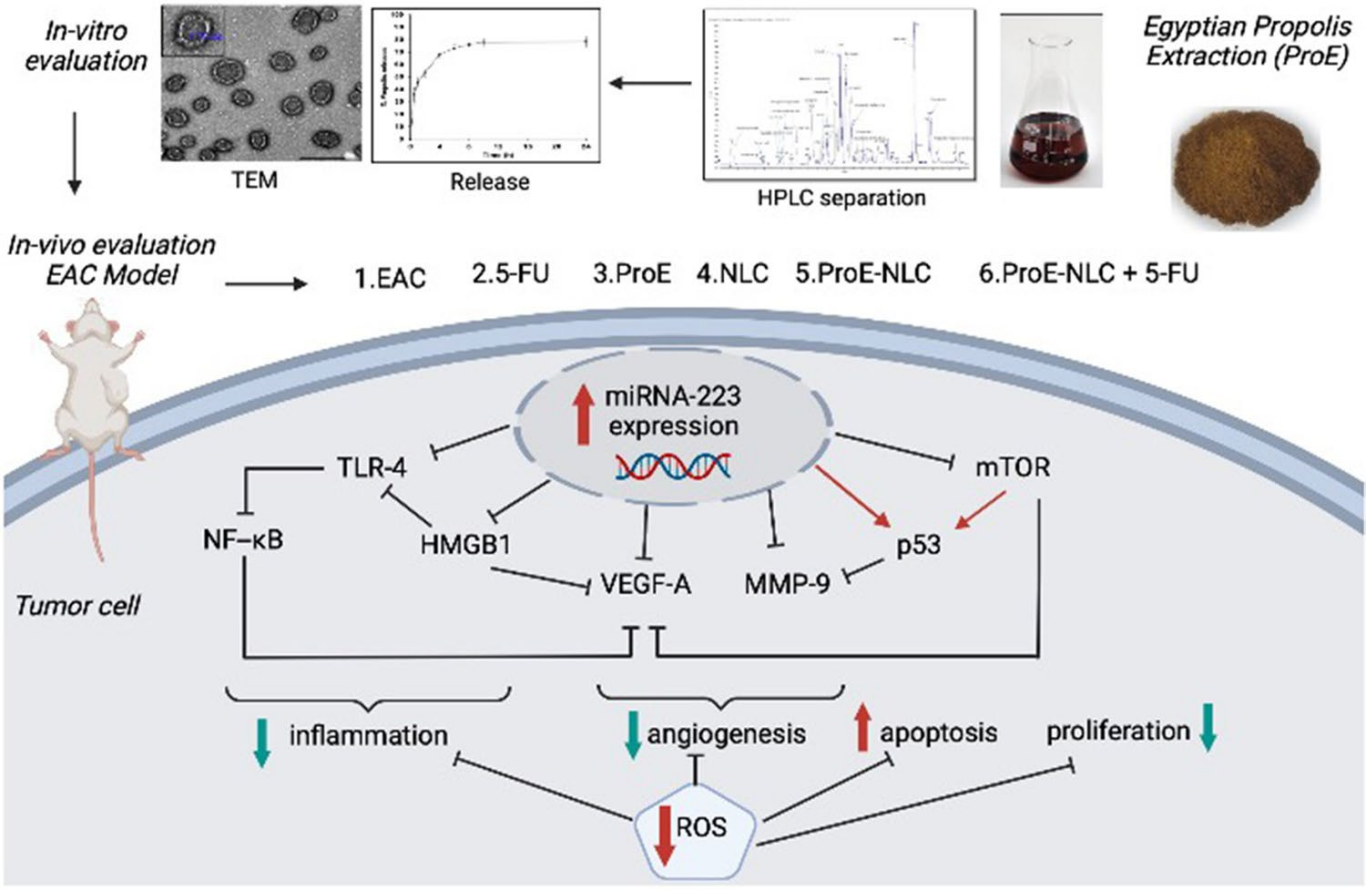
Graphical representation of the experimental design and the interrelated molecular pathways investigated in the study.

Conclusively, ProE-NLC represented a novel delivery system with excellent physicochemical properties and good stability. The proposed formulation provided a sustained drug release; thus, it is predicted to enhance the anti-tumor activity of propolis extract. ProE-NLC has potential anti-breast cancer activity in EAC-bearing mice through multiple interrelated mechanisms including, the elevation of antioxidant status, suppressing angiogenesis, inflammatory and mTOR pathways, and inducing the apoptotic pathway, all of which are a function of increased miRNA-223 expression (Fig. 7). The ProE-NLC also increased the effectiveness of cytotoxic drugs, nominating ProE-NLC as an antitumor agent against breast cancer or adjuvant treatment to cytotoxic chemotherapeutics to enhance their antitumor activity and decrease their side effects.

The future directions for the potential clinical use of ProE-NLC for breast cancer treatment include; further studies concerning the absorption, scaling up production, long-term stability, in addition to substantial in-vivo and toxicological studies to ensure safety. Testing the combination of ProE-NLC with other cytotoxic chemotherapeutics is also recommended. Thus, it promises to play an essential role as an alternative or adjuvant to chemotherapy in future breast cancer treatment and to improve patient’s quality of life.

### Supplementary Information

Supplementary Information 1.

Supplementary Information 2.

## Data Availability

All data generated or analysed during this study are included in this published article (and its Supplementary Information files).

## Abbreviations

ALT: Alanine aminotransferase
AST: Aspartate aminotransferase
CAT: Catalase
Da: Dalton
DMSO: Dimethyl sulfoxide
EAC: Ehrlich ascites carcinoma
EE: Entrapment efficiency
5-FU: 5-Fluorouracil
GR: Glutathione reductase
GSH: Reduced glutathione
HMBG1: High mobility group box 1
HR: High resolution
LC–MS/MS: Liquid Chromatography Tandem Mass Spectrometry
MDA: Malondialdehyde
MMP-9: Matrix metalloproteinase-9
mTOR: Mammalian target of rapamycin
NF-κB: Nuclear factor kappa B
NLC: Nanostructured lipid carriers
PdI: Polydispersity index
PS: Particle size
ProE: Propolis extract
P ATO 5: Precirol^®^ ATO 5
P 188: Kolliphor^®^ P 188
P 407: Kolliphor^®^ P 407
PBS: Phosphate buffer
SOD: Superoxide dismutase
TEM: Transmission electron microscope
TLR4: Toll-like receptor 4
VEGF: Vascular endothelial growth factor
ZP: Zeta potential
