# Supplementary material for: Propolis-loaded nanostructured lipid carriers halt breast cancer progression through miRNA-223 related pathways: an in-vitro/in-vivo experiment
Source: Sci Rep. 2023 Sep 21;13:15752. doi: 10.1038/s41598-023-42709-7 (PMC10514043; doi:10.1038/s41598-023-42709-7)
Supplement: Supplementary file 1 — Supplementary Information 1. [file 41598_2023_42709_MOESM1_ESM.docx]

**Determination of malondialdehyde (MDA) as thiobarbituric acid reactive substances (TBARS)**

Malondialdehyde was determined according to the method of Draper and Hadley. The tissue samples are heated with thiobarbituric acid (TBA) at low pH. The resulting pink chromogen has a maximal absorbance at 532 nm [1].

**Determination of superoxide dismutase (SOD) activity**

In the assay, xanthine is converted to superoxide radical ions, uric acid, and hydrogen peroxide by xanthine oxidase (XO). Superoxide reacts with SOD-Orange™ to generate a product that absorbs around 560 nm. SOD inhibits the reaction of SOD Orange™ with superoxide, thus reducing the absorption at 560 nm. The reduction in the absorption of SOD-Orange™ at 560 nm is proportional to SOD activity [2].

**Determination of catalase (CAT) activity**

The CAT assay takes advantage of the fact that H_2_O_2_ has an ultraviolet absorbance maximum of 240 nm. The disappearance of H_2_O_2_ caused by the action of catalase can be monitored with respect to time. The rate of decrease is a measure of the amount of catalase that catalyzes the following reaction [3].

**Determination of glutathione reductase activity**

Glutathione Reductase catalyzes the NADPH-dependent reduction of oxidized glutathione (GSSG) to reduced glutathione (GSH), which plays an important role in the GSH redox cycle that maintains adequate levels of reduced GSH. GR was assayed according to the method of [4].In the assay, GR reduces GSSG to GSH, which reacts with 5,5′-Dithiobis (2-nitrobenzoic acid) (DTNB) to generate TNB that is monitored spectrophotometrically at 412 nm. One unit of enzyme activity is defined as the amount of the enzyme that catalyzes the formation of 1µmol of TNB per minute.

**Determination of reduced glutathione content**

Reduced glutathione (rGSH) can react with Dinitrobenzoic acid (DNTB) to form a yellow complex which can be detected by colorimetric assay at 405 nm and calculate the reduced GSH content indirectly [5].

**Determination of total antioxidant capacity**

Total Antioxidant Capacity Assay Kit can measure either the combination of both small molecule antioxidants and proteins or small molecules alone in the presence of the proprietary Protein Mask. Cu2+ ion is converted to Cu+ by both small molecules and protein. The Protein Mask prevents Cu2+ reduction by protein, enabling the analysis of only the small molecule antioxidants. The reduced Cu+ ion is chelated with a colorimetric probe giving a broad absorbance peak around OD 570 nm, proportional to the total antioxidant capacity [6].

**Determination of Total Protein**

A modification method of Lowry *et al.* was used for the determination of protein in the samples. The color produced is thought to be due to a complex between the alkaline copper-phenol reagent and tyrosine and tryptophan residues of the protein in the sample. The protein concentration in each sample was calculated from the bovine serum albumin (BSA) standard curve [7].

1. Draper, H.H. and M. Hadley, *Malondialdehyde determination as index of lipid peroxidation.* Methods Enzymol, 1990. **186**: p. 421-31.

2. Marklund, S. and G. Marklund, *Involvement of the superoxide anion radical in the autoxidation of pyrogallol and a convenient assay for superoxide dismutase.* Eur J Biochem, 1974. **47**(3): p. 469-74.

3. Aebi, H., *Catalase in vitro.* Methods Enzymol, 1984. **105**: p. 121-6.

4. Smith, I.K., T.L. Vierheller, and C.A. Thorne, *Assay of glutathione reductase in crude tissue homogenates using 5,5'-dithiobis(2-nitrobenzoic acid).* Anal Biochem, 1988. **175**(2): p. 408-13.

5. Zitka, O., et al., *Redox status expressed as GSH:GSSG ratio as a marker for oxidative stress in paediatric tumour patients.* Oncol Lett, 2012. **4**(6): p. 1247-1253.

6. Rice-Evans, C. and N.J. Miller, *Total antioxidant status in plasma and body fluids.* Methods Enzymol, 1994. **234**: p. 279-93.

7. Lowry, O.H., et al., *Protein measurement with the Folin phenol reagent.* J Biol Chem, 1951. **193**(1): p. 265-75.
