# Supplementary material for: Propolis-loaded nanostructured lipid carriers halt breast cancer progression through miRNA-223 related pathways: an in-vitro/in-vivo experiment
Source: Sci Rep. 2023 Sep 21;13:15752. doi: 10.1038/s41598-023-42709-7 (PMC10514043; doi:10.1038/s41598-023-42709-7)
Supplement: Supplementary file 2 — Supplementary Information 2. [file 41598_2023_42709_MOESM2_ESM.docx]

| Groups | | Body weight (g)  At beginning of treatment | Body weight (g)  At end of treatment |
| --- | --- | --- | --- |
| NC | | 25.1±1.3 | 28.3±1.3 |
| EAC bearing mice | EAC | 21.4±2.2 | 20.6±2.7 |
|  | 5-FU treated | 24±2.5 | 24.4±1.4 |
|  | ProE treated | 22.6±1.5 | 24.2±1.4 |
|  | NLC treated | 22.5±1.8 | 22.2±1.9 |
|  | ProE-NLC treated | 23.8±2.2 | 25.6±2.6 |
|  | ProE-NLC + 5-FU | 22.4±1.8 | 23±1.3 |

**Table showing the effect of treatment on body weight.**
